# A single-dose F1-based mRNA-LNP vaccine provides protection against the lethal plague bacterium

**DOI:** 10.1126/sciadv.adg1036

**Published:** 2023-03-08

**Authors:** Edo Kon, Yinon Levy, Uri Elia, Hila Cohen, Inbal Hazan-Halevy, Moshe Aftalion, Assaf Ezra, Erez Bar-Haim, Gonna Somu Naidu, Yael Diesendruck, Shahar Rotem, Nitay Ad-El, Meir Goldsmith, Emanuelle Mamroud, Dan Peer, Ofer Cohen

**Affiliations:** ^1^ Laboratory of Precision NanoMedicine, Shmunis School for Biomedicine and Cancer Research, George S. Wise Faculty of Life Sciences, Tel Aviv University, Tel Aviv 69978, Israel.; ^2^ Center for Nanoscience and Nanotechnology, Tel Aviv University, Tel Aviv 69978, Israel.; ^3^ Department of Materials Sciences and Engineering, Iby and Aladar Fleischman Faculty of Engineering, Tel Aviv University, Tel Aviv 69978, Israel.; ^4^ Cancer Biology Research Center, Tel Aviv University, Tel Aviv 69978, Israel.; ^5^ Department of Biochemistry and Molecular Genetics, Israel Institute for Biological Research, Ness-Ziona 76100, Israel.

## Abstract

Messenger RNA (mRNA) lipid nanoparticle (LNP) vaccines have emerged as an effective vaccination strategy. Although currently applied toward viral pathogens, data concerning the platform’s effectiveness against bacterial pathogens are limited. Here, we developed an effective mRNA-LNP vaccine against a lethal bacterial pathogen by optimizing mRNA payload guanine and cytosine content and antigen design. We designed a nucleoside-modified mRNA-LNP vaccine based on the bacterial F1 capsule antigen, a major protective component of *Yersinia pestis*, the etiological agent of plague. Plague is a rapidly deteriorating contagious disease that has killed millions of people during the history of humankind. Now, the disease is treated effectively with antibiotics; however, in the case of a multiple-antibiotic-resistant strain outbreak, alternative countermeasures are required. Our mRNA-LNP vaccine elicited humoral and cellular immunological responses in C57BL/6 mice and conferred rapid, full protection against lethal *Y. pestis* infection after a single dose. These data open avenues for urgently needed effective antibacterial vaccines.

## Teaser

mRNA-LNPs vaccine provides full protection against a lethal bacterium.

## INTRODUCTION

Messenger RNA (mRNA) lipid nanoparticle (LNP) vaccines have recently been recognized as a breakthrough vaccine platform because of their effectiveness in the fight against the coronavirus disease 2019 (COVID-19) pandemic (*1*). However, while a multitude of mRNA vaccines are designed against viral diseases or cancer, bacterial pathogens remain largely untapped, with few studies demonstrating antibacterial effects (*2*–*8*). mRNA-LNPs can serve as an important tool in the arsenal toward this goal because they are clinically relevant, rapidly manufactured, and inherently modular. In the recent COVID-19 pandemic, the first mRNA-LNP vaccine entered clinical trials as soon as 63 days after viral sequence identification, emphasizing the importance of this platform in emergency preparedness (*9*).

Plague is an infectious disease caused by the Gram-negative bacterium *Yersinia pestis*, which has claimed the lives of millions of people throughout human history (*10*). Because of its lethality and infectivity, *Y. pestis* is classified as a potential bioterror agent (*11*). Plague morbidity and mortality rates have substantially decreased since the introduction of antimicrobials, but the isolation of *Y. pestis* strains resistant to multiple therapeutic antibiotic (*12*, *13*) and the fear of a natural or intentional disease outbreak initiated by antibiotic-resistant strains emphasize the need to develop vaccines against this deadly disease. Although several vaccine candidates confer protection in animal models of plague and in clinical studies, none of them has been approved for use in Western countries (*14*). An important component of these anti-plague subunit vaccines is the Caf1 (F1) capsule antigen that is expressed from the *caf1* operon on the pMT-1 103Kb plasmid of *Y. pestis* (*15*, *16*). The expression of the *caf1* operon is optimal at 37°C, i.e., upon the translocation of the pathogen from the flea vector to the mammalian host (*17*). In the course of its biogenesis, F1 polymers are generated in the periplasm of *Y. pestis* with the assistance of the Caf1M chaperon (*18*, *19*). In a subsequent step, these polymers are exported by the Caf1A usher protein to the surface of the bacterium to form a gel-like capsule that enables the pathogen to evade phagocytosis (*20*). Recombinant F1 (rF1) was readily purified from *Escherichia coli* cultures harboring the entire *caf1* operon, to produce polymeric F1, or only the circular permutated *caf1* (cp*-caf1*) gene encoding for monomeric F1 (mF1) (*21*, *22*). Recombinant polymeric F1 and mF1 are highly immunogenic and, upon vaccination, provide immunity in rodent plague models (*21*–*23*). In this study, we designed several mRNA vaccine versions based on the *Y. pestis* F1 capsule protective antigen, evaluated the antigen-specific humoral and cellular responses in mice, and assessed protection effectiveness against a fully virulent *Y. pestis* strain using challenge trials.

## RESULTS

### Design of a mF1-coding mRNA-LNPs

**Fig. 1.**
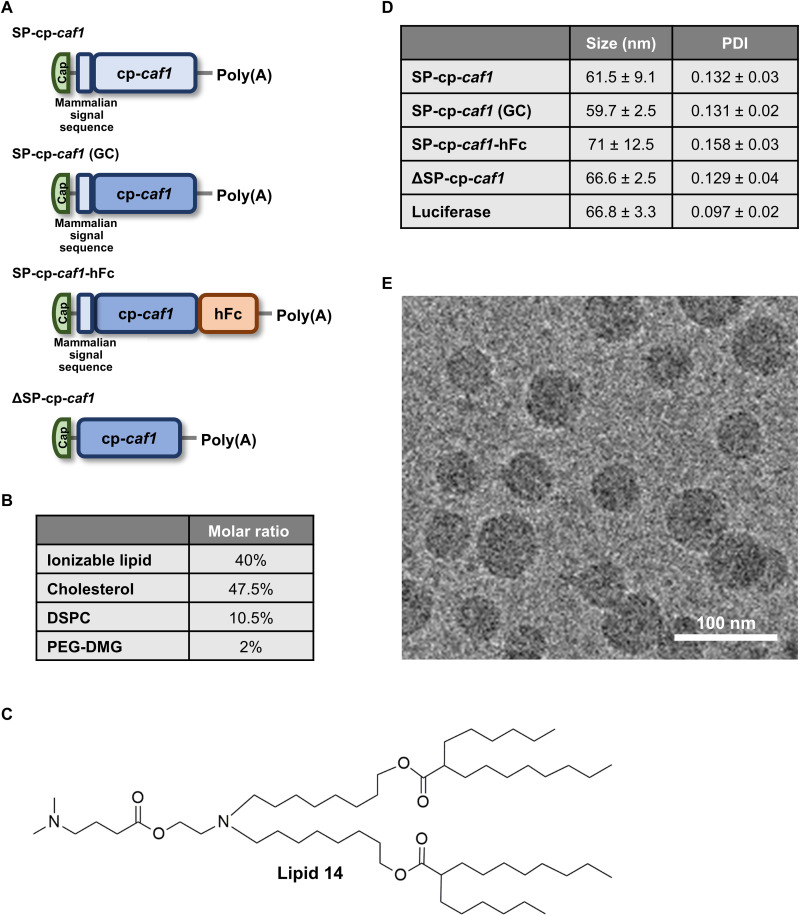
Construct design, physicochemical characterization of mRNA-LNPs formulations used throughout the study, and immunological responses elicited by SP-cp-*caf1* mRNA-LNPs. (**A**) Schematic representation of mRNA constructs used throughout the study. Elements encoded are the following: mammalian signal peptide sequence (SP), circular permutated (cp), *caf1* sequence (*caf1*), high guanine and cytosine (GC) content (GC), and human Fc sequence (hFc). Poly(A), polyadenylate. (**B**) LNP formulation used throughout the study. DSPC, distearoyl-*sn*-glycero-3-phosphocholine; PEG-DMG, dimyristoyl-rac-glycero-3-methoxypolyethylene glycol. (**C**) Chemical structure of the proprietary ionizable lipid—lipid 14. (**D**) Table summarizing LNP physicochemical aspects. (**E**) A representative cryo–electron microscopy (cryo-EM) image of LNP-encapsulated SP-cp-*caf1* mRNA. Scale bar, 100 nm.

Because the biogenesis of the F1 protein involves the concerted expression of a multitude of bacterial genes, which may be challenging to recapitulate in the mammalian system, we expressed a cp-*caf1* gene coding for mF1. Vaccination with the mF1 protein was shown to effectively induce anti-F1 immunoglobulin G (IgG) antibodies and provide protection in the mouse model of bubonic plague (*22*, *23*). In addition, we rationalized that, for the activation of a potent humoral immune response, the encoded protein should be directed toward the secretory pathway. Therefore, a signal peptide (SP) sequence originating from the human Ig kappa light chain was introduced upstream to the cp*-caf1* gene, replacing the native bacterial signal sequence, resulting in the SP-cp-*caf1* mRNA construct (Fig. 1A).

### Physicochemical characterization of SP-cp-*caf1* mRNA-LNPs

**Fig. 2.**
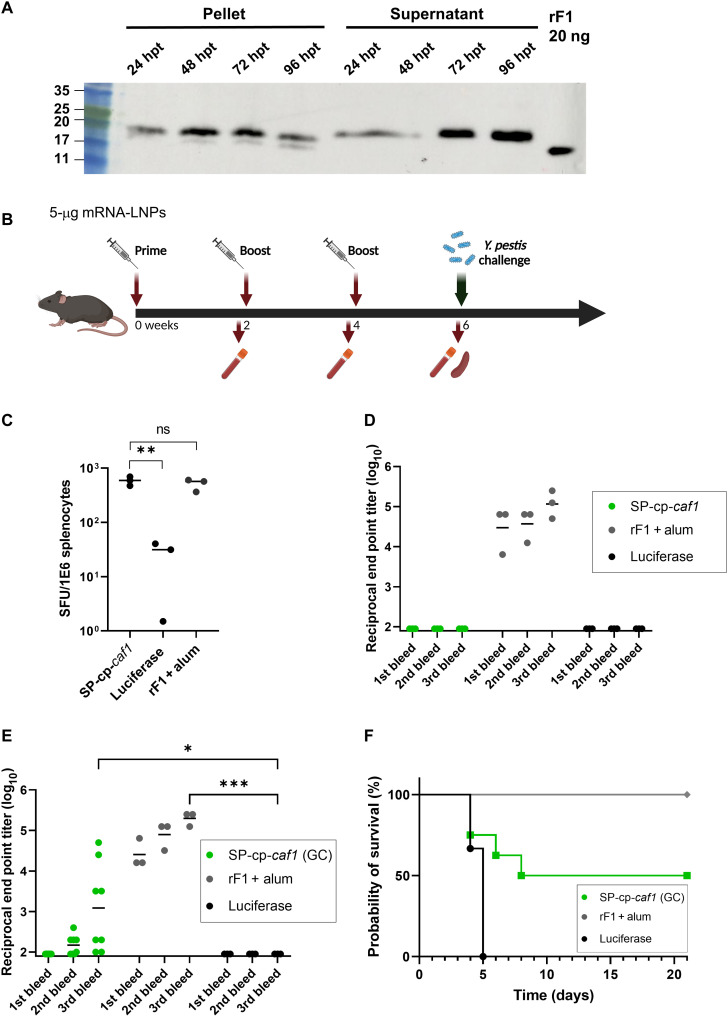
Increased GC content leads to development of anti-F1 humoral response and partially protects mice from a lethal *Y. pestis* challenge in the bubonic plague model. (**A**) Western blot analysis of F1 expression in samples collected from transfected HeLa cells at different hours posttransfection (hpt). (**B**) Schematic diagram of immunization, sample collection, and challenge. (**C**) F1-specific cellular response determined by enzyme-linked immunosorbent spot (ELISpot). (**D** and **E**) Anti-F1 IgG titers determined by enzyme-linked immunosorbent assay (ELISA). (**F**) Survival curve of C57BL/6 mice vaccinated with three doses of SP-cp-*caf1* (GC), rF1 + alum, or luciferase. Statistical analysis was performed using a one-way analysis of variance (ANOVA) followed by post hoc Newman-Keuls test (ELISpot data), a two-way ANOVA with Tukey’s multiple comparisons test (*P < 0.05, **P < 0.01, and ***P < 0.001,) (ELISA data), or log-rank (Mantel-Cox) test (for survival plot). ns, not significant.

To determine our mRNA constructs’ vaccine potential, we encapsulated the SP-cp-*caf1* mRNA in a previously reported LNP formulation that we demonstrated to be highly effective as an mRNA-LNP vaccine (Fig. 1, B and C) (*24*, *25*). Physicochemical characterization of SP-cp-*caf1* mRNA-LNPs resulted in an average size of 61.5 nm, and a polydispersity index (PDI) of 0.132 (Fig. 1D). The uniform size distribution was corroborated by cryo–electron microscopy (cryo-EM) analysis (Fig. 1E). Gel electrophoresis was used to evaluate mRNA integrity and encapsulation in LNPs (fig. S1). Transfection of SP-cp-*caf1* mRNA-LNPs into HeLa cells was used to evaluate the expression of the mF1 protein. Both supernatant and cell pellets were found to contain F1, confirming that the protein was expressed and secreted in the mammalian system (Fig. 2A).

### Evaluation of SP-cp-*caf1* mRNA-LNP immunogenicity

Next, we assessed the in vivo immunogenicity of the SP-cp-*caf1* mRNA-LNPs. Mice were intramuscularly immunized thrice, 2 weeks apart, with 5 μg of SP-cp-*caf1* mRNA-LNPs (Fig. 2B). Two weeks following administration of the third vaccine dose of SP-cp-*caf1* mRNA-LNPs or rF1, which serve as a control, a robust and statistically significant cellular immune response was determined. In contrast, humoral response was recorded only in rF1-vaccinated mice (Fig. 2, C and D). The previous studies we conducted demonstrated that animals that do not develop anti-F1 IgG antibody titers succumb rapidly to bubonic plague infection (*23*). In an attempt to increase the immunogenicity of SP-cp-*caf1* mRNA-LNPs, we hypothesized that incorporation of 1,2-Dioleoyl-3-trimethylammonium propane (DOTAP), a cationic lipid that induces strong immediate inflammatory responses via innate signaling, in the LNPs formulation might elicit humoral response in vaccinated animals (*26*). To this end, we incorporated DOTAP at 10% of the final formulation and vaccinated mice thrice with these SP-cp-*caf1* mRNA-LNPs. Not only did the vaccinated animals not develop a humoral anti-F1 response but they also demonstrated a reduced cellular response to the encoded antigen (fig. S2).

### Immunogenicity and protective efficacy of high guanine and cytosine content SP-cp-*caf1* mRNA-LNPs against a lethal *Y. pestis* challenge

Enrichment of guanine and cytosine (GC) content has been previously shown to increase mRNA levels and protein expression (*27*, *28*). We therefore hypothesized that a high GC content mRNA construct would be more potent in eliciting anti-F1 humoral responses, compared to the native *caf1* sequence encoded in the SP-cp-*caf1* mRNA. We designed a high (66%) GC content SP-cp-*caf1* mRNA construct [SP-cp-*caf1* (GC)] (Fig. 1A), encoding for the same amino acid sequence as the original SP-cp-*caf1* mRNA construct (45% GC). To evaluate the in vivo immunogenicity of the high GC content mRNA construct, mice were immunized thrice, 2 weeks apart, with 5 μg of SP-cp-*caf1* (GC) mRNA-LNPs. rF1 protein and luciferase mRNA-LNP–vaccinated animals served as positive and negative control groups, respectively. Mice vaccinated with SP-cp-*caf1* (GC) mRNA-LNPs developed low anti-F1 IgG titers (<500) after both prime and first booster vaccinations. However, administration of a second booster vaccination resulted in the elicitation of high antibody titers in 50% (four of eight) of SP-cp-*caf1* (GC) mRNA-LNP–administered animals. As expected, high anti-F1 IgG titers were recorded in rF1-vaccinated mice, and no response was found in luciferase mRNA-LNP–vaccinated animals (Fig. 2E). Considering the strong humoral response detected in 50% of SP-cp-*caf1* (GC) mRNA-LNP–vaccinated animals, we hypothesized that these animals would be able to withstand a lethal *Y. pestis* challenge in the bubonic plague mouse model. Therefore, 2 weeks after the last booster vaccination, all animals were inoculated subcutaneously with a lethal dose of the fully virulent *Y. pestis* strain Kimberley53 (Kim53) and were monitored for survival. Vaccination with SP-cp-*caf1* (GC) mRNA-LNPs conferred 50% protection (four of eight mice) against the lethal challenge. As expected, all surviving mice exhibited high anti-F1 titers before the challenge, demonstrating a correlation between anti-F1 titers and survival probability.

### Design and evaluation of human Fc-conjugated and SP-devoid *caf1* mRNA constructs

In an effort to improve the vaccine’s effectiveness, we adopted two different approaches to express mF1 in the mammalian system, based on the SP-cp-*caf1* (GC) mRNA construct. First, we designed a human Fc-conjugated SP-cp-*caf1* (SP-cp-*caf1*-hFc) construct (Fig. 1A) based on the rationale that hFc conjugation was reported to increase the stability, half-life, and immunogenicity of the target protein (*29*–*31*). Second, we designed an mRNA construct encoding a cytoplasmic mF1 antigen by removing the mammalian SP from the mRNA sequence (ΔSP-cp-*caf1*) (Fig. 1A). This approach was based on our hypothesis that the passage of the mF1 via the eukaryotic secretory pathway may result in reduced immunogenicity due to a variety of naturally occurring posttranslational modifications (PTMs) (*32*). Both ΔSP-cp-*caf1* and SP-cp-*caf1-*hFc were designed with a high GC content similar to the SP-cp-*caf1* (GC) sequence.

### Immunogenicity and protective efficacy of ΔSP-cp-*caf1* and SP-cp-*caf1*-hFc mRNA-LNPs against a lethal *Y. pestis* challenge

**Fig. 3.**
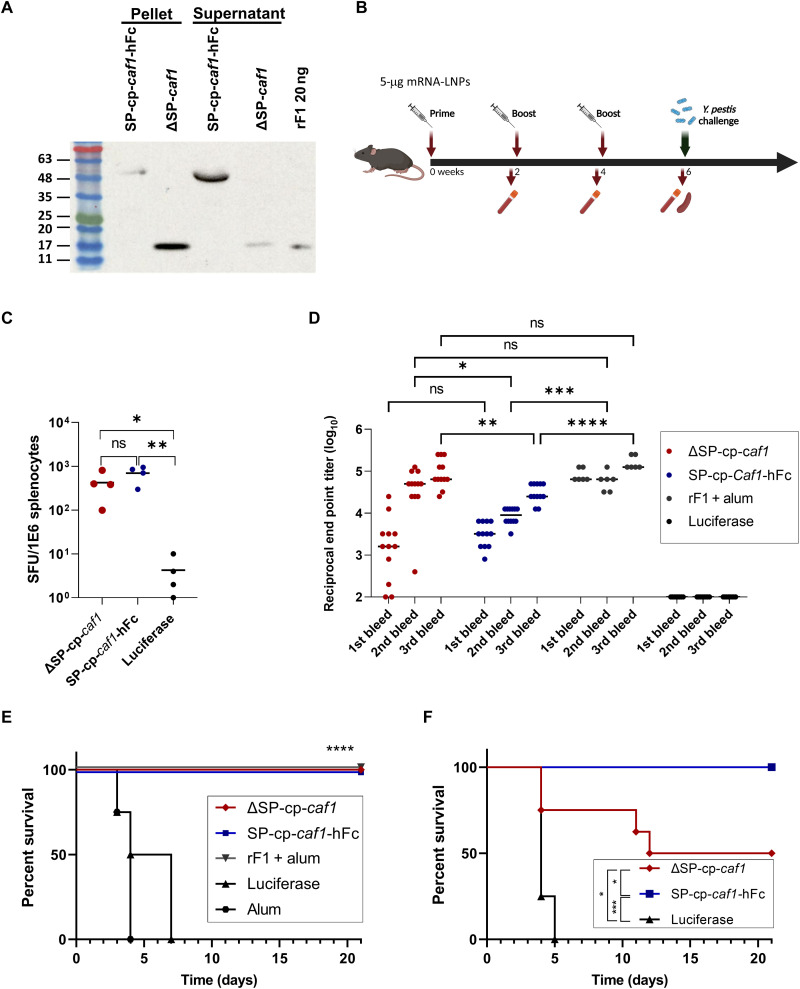
SP-cp-*caf1*-hFc and ΔSP-cp-*caf1* mRNA LNPs elicit robust cellular and humoral immune responses and protect mice against a lethal *Y. pestis* challenge in the bubonic plague model. (**A**) Western blot analysis of F1 expression in HeLa cells. (**B**) Schematic diagram of immunization and sample collection. (**C**) F1-specific cellular response determined by ELISpot. (**D**) Anti-F1 IgG titers determined by ELISA. (**E**) Survival curve of C57BL/6 mice vaccinated with three doses of ΔSP-cp-*caf1*, SP-cp-*caf1-*hFc, rF1 + alum, luciferase, or alum alone. (**F**) Survival curve of C57BL/6 mice vaccinated with a single dose of ΔSP-cp-*caf1*, SP-cp-*caf1-*hFc, or luciferase. Statistical analysis was performed using a one-way ANOVA followed by post hoc Newman-Keuls test, two-way ANOVA with Tukey’s multiple comparisons test (*P < 0.05, **P < 0.01, ***P < 0.001, and ****P < 0.0001) (for immune responses) or log-rank (Mantel-Cox) test (****P < 0.0001) (for survival plot).

The following step was to evaluate the immune responses elicited by SP-cp-*caf1*-hFc and ΔSP-cp-*caf1*–based mRNA-LNP vaccines. SP-cp-*caf1*-hFc and ΔSP-cp-*caf1* mRNAs were encapsulated in the lipid formulation previously described (Fig. 1, B and C), and protein expression was confirmed in vitro (Fig. 3A). As expected, mF1 expressed from the SP-cp-*caf1*-hFc mRNA construct was detected mostly in the supernatant fraction, whereas mF1 encoded by ΔSP-cp-*caf1* was expressed predominantly in the cellular lysate fraction, confirming the inability of the protein to enter the secretory pathway (Fig. 3A).

To evaluate the in vivo immunogenicity of the mF1 expressed from SP-cp-*caf1*-hFc and ΔSP-cp-*caf1* mRNA-LNPs, mice were immunized thrice, 2 weeks apart, with 5 μg of each construct (Fig. 3B). Antigen-specific cellular responses were recorded following vaccination with both SP-cp-*caf1*-hFc and ΔSP-cp-*caf1* mRNA-LNPs (Fig. 3C), similar to the original SP-cp-*caf1* construct. However, immunization with both SP-cp-*caf1*-hFc and ΔSP-cp-*caf1* mRNA-LNPs led to a robust, time-dependent anti-F1 humoral response. A different trend was observed in F1 antibody titers recorded in the two vaccination groups. While ΔSP-cp-*caf1* mRNA-LNP–vaccinated animals exhibited a wide range of anti-F1 titers (100 to 25,000) after the first vaccine administration, a more uniform pattern of anti-F1 titers was detected in SP-cp-*caf1*-hFc–vaccinated mice (1600 to 6400), and there was no statistically significant difference between the groups. Administration of a second vaccination (first booster) led to a notable increase in recorded titers, with a more pronounced boosting effect in the ΔSP-cp-*caf1* mRNA-LNP group and 90% of animals showing titers of 25,000 to 125,000. The overall average anti-F1 titer after the second and third administration (first and second booster) was significantly higher in ΔSP-cp-*caf1* mRNA-LNPs compared to SP-cp-*caf1*-hFc–vaccinated mice and compared to rF1-vaccinated mice. Encouraged by the high anti-F1 IgG antibody titers and strong cellular response recorded in both ΔSP-cp-*caf1*– and SP-cp-*caf1*-hFc mRNA-LNP–immunized mice, we hypothesized that the vaccinated animals would be able to withstand a lethal *Y. pestis* challenge. Consequently, all vaccinated animals were inoculated subcutaneously with the fully virulent *Y. pestis* strain Kim53 [100 median lethal dose (LD_50_)]. Encouragingly, both ΔSP-cp-*caf1* and SP-cp-*caf1*-hFc mRNA-LNP immunizations were able to confer full protection against the lethal challenge (Fig. 3E). In accordance with previous reports (*21*, *33*), all negative control mice succumbed to the infection by day 7, whereas all rF1 + alum-vaccinated animals survived the lethal challenge.

### Protective efficacy of a single dose of SP-cp-*caf1*-hFc mRNA-LNPs against a lethal *Y. pestis* challenge

Considering the high effectiveness of the two mRNA-LNPs following a three-dose vaccination regimen and the humoral response observed 2 weeks after the first administration, we next sought to investigate whether a single-vaccination regimen would be sufficient to provide prophylactic protection against a lethal *Y. pestis* challenge. To this end, we vaccinated mice once with 5 μg of each mRNA-LNP construct and subcutaneously challenged them 2 weeks later with *Y. pestis* Kim53 (100 LD_50_). In accordance with the high and uniform anti-F1 IgG titers previously generated by the SP-cp-*caf1*-hFc mRNA-LNP formulation, all mice in this group survived the challenge (Fig. 3F). Mice vaccinated once with ΔSP-cp-*caf1* mRNA-LNPs were only 50% protected as reflected by the more heterogenous anti-F1 IgG titers generated by this formulation 2 weeks after the administration of the vaccine (Fig. 3D). As expected, all control mice vaccinated with luciferase mRNA-LNP succumbed to the infection by day 5.

## DISCUSSION

In this study, we describe the successful utilization of the mRNA-LNP vaccine platform for protection against the highly virulent bacterial pathogen *Y. pestis*. In contrast to viral proteins, which are naturally adapted for expression by eukaryotic systems, the construction of an mRNA vaccine platform that encodes for the expression of a bacterial protein that will generate protective immunity requires adaptations. Thus, to enable the efficient expression, translocation, and secretion of the F1 outer membrane protein by the eukaryotic mammalian system, we replaced the F1 prokaryotic secretion signal with a eukaryotic one (Fig. 1A). In the natural *Y. pestis* expression environment, the newly synthesized F1 protein is translocated to the periplasm of the bacterial cell, where it is properly folded by the Caf1M chaperon and exported to the outer surface by the Caf1A usher protein, resulting in high–molecular weight F1 oligomers (*34*). Because these elements do not exist in the eukaryotic expression system, we expressed the F1 protein as a monomer using the circular permutated form of the protein that was previously shown to be protective in a bubonic plague mouse model (*22*, *23*). Regarding the translatability of these results from mice to humans, anti-F1 IgG titers highly correlate both in humans and in mice to the ability to resist infection (*35*). Vaccination with only F1 would not provide protection against noncapsulated (Δ*caf1*) *Y. pestis* strains; however, infection with these strains is extremely rare (*36*). In this case, co-vaccination with the low-calcium response virulence protein of *Y. pestis* is expected to provide protection against nonencapsulated strains as well (*37*).

The secreted *caf1* (SP-cp-*caf1*) mRNA-LNP vaccine yielded antigen-specific cellular responses but not humoral responses. An attempt to increase the formulation’s immunogenicity by incorporating 10% DOTAP in the LNP formulation was unsuccessful and resulted in reduced cellular response and lack of humoral response. Although DOTAP elicits rapid immediate inflammatory responses, the diminished adjuvanticity of DOTAP recorded in our study is in line with data published in a recent study, demonstrating that, in contrast to ionizable lipid-formulated LNPs, DOTAP-formulated LNPs fail to induce serum hemagglutination inhibition titers in a mouse model (*38*).

While cellular responses can be beneficial for protection against bacterial pathogens with a predominantly intracellular life cycle, such as *Mycobacterium tuberculosis* (*39*), a mandatory robust humoral response is required against virulent bacteria that are mainly extracellular, such as *Y. pestis*. To this end, we designed and evaluated three additional cp-*caf1*–based mRNA-LNP vaccines. First, we used a GC-rich mRNA construct, encoding the exact same amino acid sequence as the original, low-GC content construct. Given the reported correlation between high GC content and increased mRNA stability and level of protein expression, we hypothesized that increasing the GC content of the original SP-cp-*caf1* mRNA-LNP may yield a strong humoral response. Such beneficial effects were recorded in animals vaccinated with SP-cp-*caf1* (GC) mRNA-LNP; however, only 50% of the animals developed high IgG titers against F1, which enabled them to survive the lethal challenge with the virulent strain (Fig. 2, B and C). To further augment the humoral response elicited by the mRNA-encoded F1 antigen, we introduced two additional modifications to the SP-cp-*caf1* (GC) mRNA construct, representing different approaches for the expression of a bacterial antigen in the eukaryotic environment: an hFc-conjugated construct and a SP-devoid construct. Administration of both hFc-conjugated and SP-devoid cp-*caf1* mRNA constructs resulted in powerful antigen-specific humoral and cellular responses that protected mice from a lethal *Y. pestis* challenge.

The process of PTMs in the secretory pathway was reported to impair immune responses to nucleic acid–based vaccines (*40*) and may account for the differential immune responses observed between the SP-cp-*caf1* and ΔSP-cp-*caf1* constructs. Secretory proteins require an SP to progress through the Golgi apparatus and reach the cell surface for secretion. However, a growing body of evidence suggests that cytosolic proteins lacking an SP can be secreted via alternative routes, known collectively as unconventional protein secretion. These include inflammation and stress-induced plasma membrane pore formation, allowing proteins to translocate across the cell membrane, and also secretion by membrane-bound organelles such as endosomes and autophagosomes (*41*–*43*). These pathways are still being elucidated, and data connecting these studies to nucleic acid vaccines are scarce and, therefore, can be very interesting to explore in the context of antibacterial vaccines. Although not in the scope of our study, these mechanisms of SP-devoid protein secretion may account for the induction of specific humoral response following immunization with the ΔSP-cp-*caf1* construct.

The high efficacy of the SP-cp-*caf1*-hFc construct may be explained by several factors. Fusion of IgG-Fc domain to a target protein has been previously demonstrated to increase its half-life, immunogenicity, solubility, and delivery efficiency (*29*–*31*, *44*, *45*). Fc fusion has also been shown to have adjuvant activity, enhancing both cellular and humoral immune responses. In addition, Fc fusion facilitates correct protein folding and enhances binding to antigen-presenting cells (*46*). Now, over a dozen Fc fusion products have been approved by the Food and Drug Administration and European Medicines Agency for use in the clinic (*47*). The immunological benefits described above may account for the SP-cp-*caf1*-hFc–mediated robust humoral response, even when the expressed protein is shuttled via secretory pathways. The mechanisms underlying the differential immune responses between the mRNA constructs remain to be elucidated.

Last, we observed full protection in animals vaccinated with a single dose of SP-cp-*caf1*-hFc, while partial protection was afforded upon vaccination with ΔSP-cp-*caf1* (Fig. 3F). This trend correlates well with the anti-F1 IgG titers recorded 2 weeks after the first dose vaccination in the three-dose vaccination study (Fig. 3D). The ability of a vaccine to confer protection after a single administration is of an utmost importance. This allows rapid response to local outbreaks without the need for booster shots, therefore easier to implement logistically, requires fewer vaccine shots to be manufactured, and may be less reactogenic than a repeated vaccination regimen.

Now, most mRNA-LNP vaccines in preclinical and clinical trials are directed against viral pathogens. The few reported studies on mRNA vaccines against bacterial pathogens mostly describe moderate reduction in bacterial burden and/or inferior efficacy of the mRNA construct in comparison to the recombinant protein in survival studies. Our study demonstrates a rapid, fully protective mRNA-LNP vaccine against the lethal *Y. pestis* bacteria. The results of the current study suggest that the mRNA-LNP platform can be harnessed for development of effective vaccination against bacterial pathogens. The clinically relevant mRNA-LNP vaccine platform holds several advantages over traditional recombinant protein vaccines. First, in contrast to traditional recombinant protein vaccines, mRNA vaccines are highly versatile and can be rapidly manufactured and easily adjusted to express different antigens, allowing quick adaptation for protection against variants of concern in emerging pandemics. Next, while protein vaccines mainly elicit humoral responses, mRNA-LNP vaccines induce both humoral and cellular immune responses, expanding the potential antipathogenic effect against pathogens with both intra- and extracellular life cycles. In addition, the inherent self-adjuvanticity of both mRNA and LNP formulation can promote robust, long-lasting immune responses, without the need for an additional adjuvant.

A major limitation in mRNA-LNP vaccines is the inherent instability of mRNA molecules and the resulting requirement for cold (−20°C) or ultracold (−70°C) shipping and storage. In addition, as suggested in the current study, the expression of bacterial proteins in a mammalian system can lead to the production of proteins that may undergo different PTMs, potentially altering the immunogenicity of the original bacterial antigen. Accordingly, attention should be paid to ensuring rational and optimal design of the relevant bacterial antigen for induction of a robust and effective immune response. Therefore, we believe that the data presented in this study provides a framework for other antibacterial mRNA-LNP vaccines in the future. These findings are of substantial relevance and immense importance, considering the global emerging crisis of antibiotic resistance and the lack of effective conventional therapies and vaccine candidates.

## MATERIALS AND METHODS

### Materials

Cholesterol, distearoyl-*sn*-glycero-3-phosphocholine (DSPC), and dimyristoyl-rac-glycero-3-methoxypolyethylene glycol (PEG-DMG) were from Avanti Polar Lipids (Alabaster, AL, USA). The proprietary ionizable lipid (lipid 14) was synthesized in-house by G. S. Naidu (fig. S3) (*48*). mRNA sequences were purchased from TriLink (San Diego, CA, USA) or synthesized by an in vitro transcription (IVT) reaction using the MEGAscript T7 transcription kit and cleaned by the MEGAclear transcription clean-up kit both from Thermo Fisher Scientific (Waltham, MA, USA). All mRNA sequences were synthesized with complete N1-methyl-pseudouridine nucleotide substitution.

### Design of mRNA constructs

Four mRNA constructs encoding for the various versions of the cp-*caf1* antigen were designed as follows: The *caf1* gene (GenBank, account number X61996) section coding for Pro^37^ to Ser^168^ was fused to a linker coding for Thr-Gly-Ser-Gly-Asn-Gly followed by Ala^22^ to Glu^36^ of the *caf1* gene to generate mF1. SP-cp-*caf1* encodes for the mF1 protein, with the addition of a 5′ SP sequence originating from the Ig light chain variable region (GenBank, account number U43767.1). SP-cp-*caf1* (GC) encodes the same amino acid sequence as SP-cp-*caf1*, with an enriched GC content (66% versus 45% in SP-cp-*caf1*). ΔSP-cp-*caf1* encodes for the mF1 protein devoid of the SP sequence. SP-cp-*caf1*-hFc is composed of the SP-cp-*caf1* sequence, followed by the sequence coding for the constant region of the human IgG1 (adopted from GenBank account number AEV43323.1). Luciferase control mRNA encodes the firefly luciferase (GenBank, account number AB762768). All mRNA constructs included an initiator methionine and a Kozak consensus sequence.

### LNP preparation and characterization

Ionizable lipid, cholesterol, DSPC, and PEG-DMG were mixed at a molar ratio of 40:47.5:10.5:2 with absolute ethanol in a tube. mRNA payloads were suspended in 50 mM citrate buffer (pH 4.5). To create LNPs, a dual-syringe pump was used to transport the two solutions through the NanoAssembler micromixer from Precision NanoSystem (Vancouver, British Columbia, Canada) at a total flow rate of 12 ml/min. The particles were then transferred into dialysis overnight against PBS. Particles in PBS were analyzed for size and uniformity by dynamic light scattering. Zeta potential was determined using the Malvern Zetasizer (Malvern, Worcestershire, UK). mRNA encapsulation in LNPs was calculated according to the Quant-iT RiboGreen RNA assay kit (Thermo Fisher Scientific, Waltham, MA, USA) by calculating the percentage encapsulation at 100% − (mRNA-LNPs/mRNA-LNPs with Triton X-100). All mRNA-LNP formulations exhibited an encapsulation efficiency of >92%.

### Cell lines

HeLa cells (American Type Culture Collection, CCL-2) were maintained at 37°C, 5% CO_2_ in Dulbecco’s modified Eagle’s medium supplemented with 10% fetal bovine serum, MEM nonessential amino acids, 2 mM l-glutamine, 1 mM sodium pyruvate, penicillin (100 U/ml) and streptomycin (0.1 mg/ml) (P/S) (Biological Industries, Israel).

### Cell transfection and Western blotting

One day before the transfection, HeLa cells were seeded in 24-well plates at a density of 10^5^ cells per well. At the day of the transfection, SP-cp-*caf1*, ΔSP-cp-*caf1*, or SP-cp-*caf1*-hFc mRNA-LNPs were added to the wells, and cells were harvested at 24 to 96 hours for detection of the F1 protein in cell lysate (cellular fraction) and cell supernatant (secreted fraction). For cellular fraction evaluation, cells were washed with phosphate-buffered saline (PBS) and lysed with radioimmunoprecipitation assay lysis buffer (Merck). The concentration of protein was measured by the Bradford method, and equal amounts of protein were resuspended in the protein sample buffer with β-mercapthoethanol and boiled before separation. Thirty micrograms of cell lysate in 30 μl of buffer or 30 μl of cell supernatant in buffer was separated by 12% SDS–polyacrylamide gel electrophoresis. Gels were blotted by dry transfer with the iBlot Gel transfer device (Thermo Fisher Scientific) to iBlot mini nitrocellulose membranes. The membrane was blocked for 1 hour at room temperature with 5% skim milk (DIFCO) in phosphate-buffered saline with Tween (PBST). F1 expression was detected by overnight incubation with purified IgG fraction from serum of rabbit immunized with the rF1 protein (1:10,000 dilution), followed by incubation with a secondary horseradish peroxidase–conjugated anti-rabbit antibody (1:5000 dilution; Jackson ImmunoResearch, #111-035-003) for 1 hour at room temperature. Reactive bands were detected by development with a SuperSignal West Pico PLUS chemiluminescent substrate (Thermo Fisher Scientific) and detection by Amersham ImageQuant 800 Western blot imaging system (Cytiva Life Sciences).

### Anti-F1 enzyme-linked immunosorbent assay

Flat-bottom Maxisorp 96-well microtiter plates (Thermo Fisher Scientific) were coated with 500 ng of purified polymeric F1 [provided by the Biotechnology Department at the Israel Institute for Biological Research (IIBR)] in 50 mM carbonate-bicarbonate buffer (pH 9.6) (Sigma-Aldrich). Following a blocking step with 1× PBS, 2% bovine serum albumin, and 0.05% Tween 20, tested sera were serially diluted in twofold dilutions in a final volume of 50 μl. Alkaline phosphatase–conjugated goat anti-mouse IgG (1/2000 dilution; Sigma-Aldrich) was used as the second layer for anti-F1 IgG titer determination. All incubation steps were performed for 1 hour at 37°C. The plates were extensively washed with 1× PBS and 0.05% Tween 20 before the following step. Titers were defined as the reciprocal values of the end point serum dilutions that displayed optical density at 405 nm (OD_405_) values twofold higher than the normal serum controls obtained from naïve animals. Enzyme-linked immunosorbent assay (ELISA) analysis was performed in technical duplicates for each animal tested.

### Cryo–electron microscopy

Samples were prepared in a closed chamber at a controlled temperature and at water saturation. A 5- to 6-μl drop of each suspension was placed on a 200-mesh transmission electron microscope (TEM) copper grid covered with a perforated carbon film. The drop was blotted, and the sample was plunged into liquid ethane (−183°C) to form a vitrified specimen and then transferred to liquid nitrogen (−196°C) for storage. Vitrified specimens were examined at temperatures less than −175°C in a Talos F200C with field emission gun operated at 200 kV or a Tecnai T12 G2 TEM (FEI, Netherlands). Images were recorded on a cooled Falcon IIIEC (FEI) direct detection device by TIA software attached to the Talos or a Gatan MultiScan 791 camera by Digital Micrograph software (Gatan, UK) on the Tecnai TEM. Volta PhasePlate was used for contrast enhancement. Images are recorded in the low-dose imaging mode to minimize beam exposure and electron beam radiation damage using laboratory procedures.

### Ethics statement

This study was carried out in strict accordance with the recommendations for the Care and Use of Laboratory Animals of the National Institutes of Health. All animal experiments were performed in accordance with Israeli law and were approved by the Ethics Committee for animal experiments at the IIBR (permit numbers M-55-21 and M-14-22). During the experiments, the mice were monitored daily. Humane end points were used in our survival studies. Mice exhibiting loss of the righting reflex were euthanized by cervical dislocation.

### Animals

Female C57BL/6JOlaHsd mice (6 to 8 weeks old) were obtained from Envigo (Israel) and randomly assigned into cages in groups of 10 animals. The mice were allowed free access to water and rodent diet (Harlan, Israel).

### Animal vaccination experiments

Female C57BL/6 mice (6 to 8 weeks old) were vaccinated intramuscularly (50 μl to each hind leg muscle, for a total of 100 μl) thrice with 5 μg of SP-cp-*caf1*, SP-cp-*caf1* (GC), ΔSP-cp-*caf1*, SP-cp-*caf1*-hFc mRNA-LNPs, or luciferase mRNA-LNPs (negative control). Alum-adsorbed rF1 protein (80 μg per mouse per dose) and alum (0.36% final concentration per mouse per dose) were intramuscularly administered as positive and negative controls, respectively. The first vaccination study included the following groups: SP-cp-*caf1* mRNA-LNPs (*n* = 3), rF1 + alum (*n* = 3), and luciferase mRNA-LNPs (*n* = 3). The second vaccination study included the following groups: SP-cp-*caf1* (GC) mRNA-LNPs (*n* = 8), rF1 + alum (*n* = 3), and luciferase mRNA-LNPs (*n* = 3). The third vaccination study included the following groups: ΔSP-cp-*caf1* mRNA-LNPs (*n* = 12), SP-cp-*caf1*-hFc mRNA-LNPs (*n* = 12), rF1 + alum (*n* = 6), luciferase mRNA-LNPs (*n* = 8), and alum only (*n* = 4). Blood samples were collected from the tail vein 1 day before boost administration. Spleens and blood samples were collected 2 weeks after administration of the last booster shot for evaluation of immunological responses. For the single-dose vaccination study, C57BL/6 mice were vaccinated once with 5 μg of ΔSP-cp-*caf1* (*n* = 8), SP-cp-*caf1*-hFc (*n* = 8), or luciferase mRNA-LNPs (negative control) (*n* = 4).

### Murine interferon-γ enzyme-linked immunosorbent spot assay

Mice spleens were dissociated in gentleMACS C tubes (Miltenyi Biotec), filtered, treated with red blood cell lysing buffer (Sigma-Aldrich, no. R7757), and washed. Pellets were resuspended in 1 ml of CTL-Test medium [Cellular Technology Limited (CTL), no. CTLT 005] supplemented with 1% fresh glutamine and 1 mM P/S (Biological Industries, Israel), and single-cell suspensions were seeded into 96-well, high-protein-binding, polyvinylidene difluoride filter plates at 300,000 to 400,000 cells per well. Mice were tested individually in duplicates by stimulation with a 15-mer peptide library spanning the F1 protein (10 μg/ml) (GenScript), concanavalin A (2 μg/ml; Sigma-Aldrich, no. 0412) as positive control, or CTL medium as negative control (no antigen). Cells were incubated with antigens for 24 hours, and the frequency of interferon-γ (IFN-γ)–secreting cells was determined using a murine IFN-γ single-color enzymatic enzyme-linked immunosorbent spot (ELISpot) kit (CTL, no. MIFNG 1M/5) with strict adherence to the manufacturer’s instructions. Spot-forming units were counted using an automated ELISpot counter (CTL).

### Mouse infection

The fully virulent *Y. pestis* strain Kim53 was grown on brain-heart infused (BHI) agar plates at 28°C. Several isolated and typical colonies were suspended in saline to generate a bacterial suspension at an OD_660_ of 0.1 [equals to 1 × 10^8^ colony-forming units (CFU)/ml]. This bacterial suspension was serially diluted, and a dose of 100 CFU (100 LD_50_) was injected subcutaneously into the lower right backs of the mice. The infectious dose was verified by plating diluted bacterial suspensions onto BHI agar plates. Immunization groups were as follows: for three-dose vaccination study: SP-cp-*caf1* mRNA-LNPs (GC) (*n* = 8), ΔSP-cp-*caf1* mRNA-LNPs (*n* = 8), SP-cp-*caf1*-hFc mRNA-LNPs (*n* = 8), rF1 + alum (*n* = 6), luciferase mRNA-LNPs (*n* = 4), and alum only (*n* = 4); for single-dose vaccination study: ΔSP-cp-*caf1* mRNA-LNPs (*n* = 8), SP-cp-*caf1*-hFc mRNA-LNPs (*n* = 8), and luciferase mRNA-LNPs (*n* = 4). Survival of infected mice was daily monitored for 21 days. Sterility of surviving mice was verified by plating spleen homogenates onto BHI agar plates supplemented with streptomycin (50 μg/ml) and by incubation at 28°C for 48 hours.

### Graph schematics

Vaccine administration illustrations were created by biorender.com.

### Statistical analysis

All values are presented as means + SEM. Statistical analysis was performed using either a two-way analysis of variance (ANOVA) with Tukey’s multiple comparisons test (for ELISA data) or a one-way ANOVA followed by post hoc Newman-Keuls test (for ELISA and ELISpot data) or log-rank (Mantel-Cox) test (for survival data) (*P < 0.05, **P < 0.01, ***P < 0.001, and ****P < 0.0001). All statistical analyses were performed using GraphPad Prism 8 statistical software.

## Notes

View/request a protocol for this paper from *Bio-protocol*.
