# Supplementary material for: A single-dose F1-based mRNA-LNP vaccine provides protection against the lethal plague bacterium
Source: Sci Adv. 2023 Mar 8;9(10):eadg1036. doi: 10.1126/sciadv.adg1036 (PMC9995031; doi:10.1126/sciadv.adg1036)
Supplement: Supplementary file 1 — Figs. S1 to S3 [file sciadv.adg1036_sm.pdf]

Science Advances
AAAS

Supplementary Materials for

A single-dose F1-based mRNA-LNP vaccine provides protection against the lethal plague bacterium

Edo Kon *et al.*

Corresponding author: Dan Peer, peer@tauex.tau.ac.il

Sci. Adv. **9**, eadg1036 (2023)
DOI: 10.1126/sciadv.adg1036

This PDF file includes:

Figs. S1 to S3

Supplementary Materials

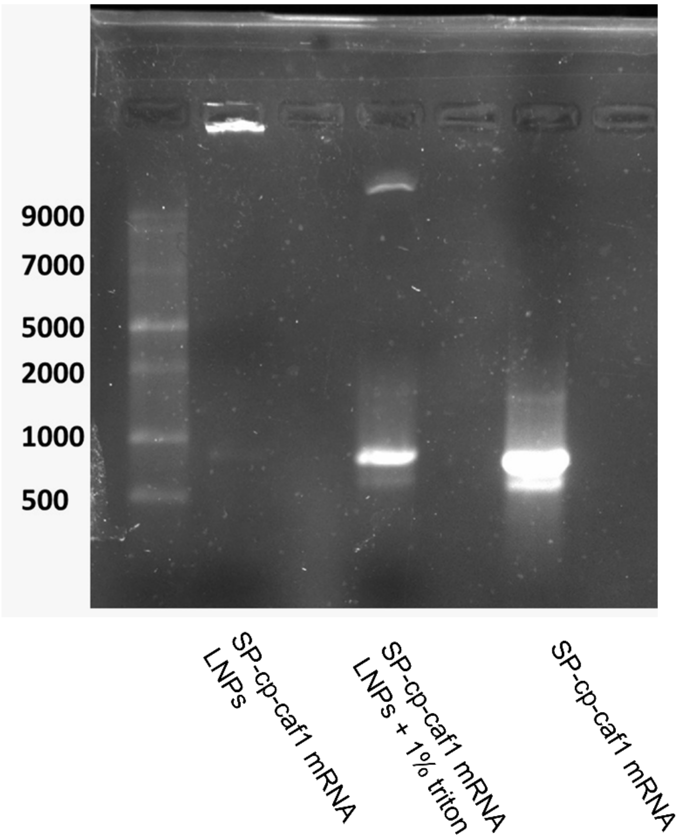

Fig. S1. mRNA LNPs evaluation by agarose gel electrophoresis. mRNA LNPs were co-incubated with 1% triton for 15 minutes at 37ºC. After incubation, samples were separated by a 2% agarose TBE gel.

A

	Molar Ratio
**Lipid 14**	36%
**DOTAP**	10%
**Cholesterol**	42.75%
**DSPC**	9.45%
**PEG-DMG**	1.8%

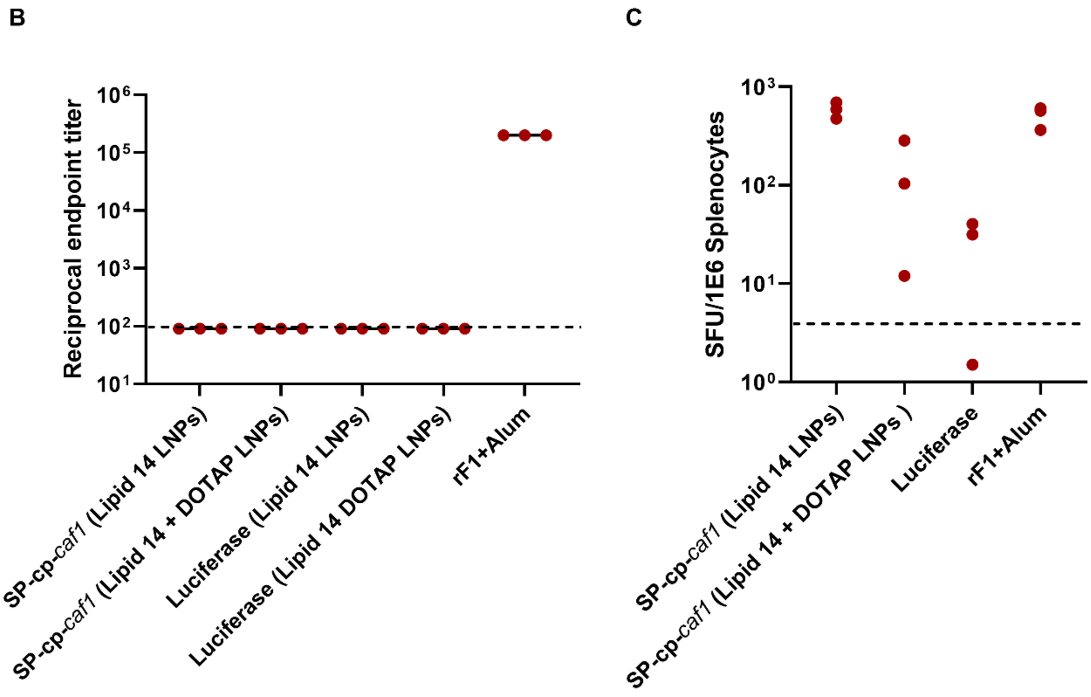

Fig. S2. Vaccination with SP-cp-*caf1* mRNA-LNPs formulated with DOTAP. (**A**) SP-cp-*caf1* mRNA-LNPs with DOTAP formulation. (**B**) Anti-F1 IgG titers determined by ELISA after three administrations. (**C**) F1-specific cellular response determined by ELISpot after three administrations.

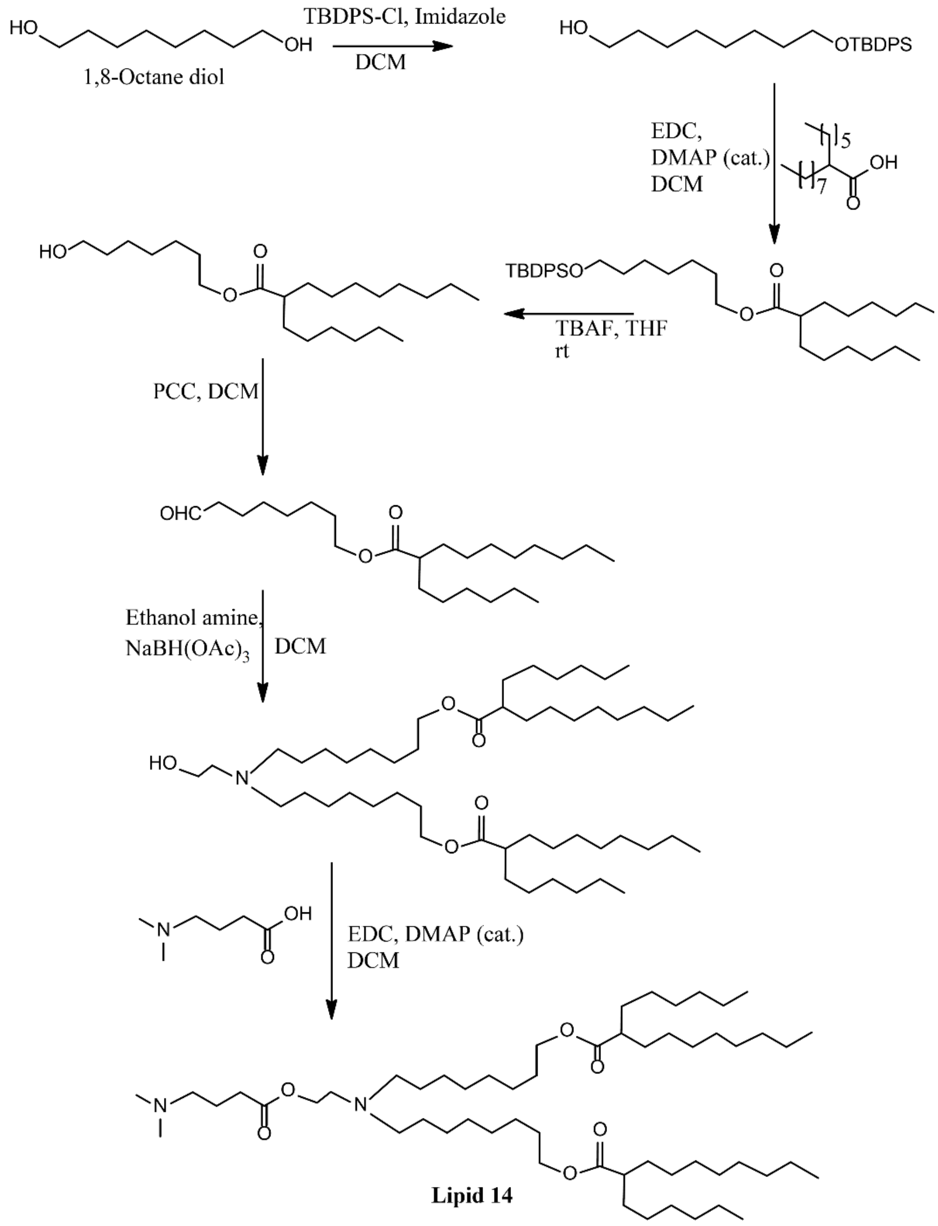

Fig. S3. Synthesis of lipid 14.
